# Role of interleukin-12 gene polymorphisms in the onset risk of cancer: a meta-analysis

**DOI:** 10.18632/oncotarget.16080

**Published:** 2017-03-10

**Authors:** Yi Zheng, Meng Wang, Tian Tian, Kang Liu, Xinghan Liu, Yajing Zhai, Shuai Lin, Pengtao Yang, Shanli Li, Zhijun Dai, Jun Lu

**Affiliations:** ^1^ Clinical Research Center, First Affiliated Hospital of Xi’an Jiaotong University, Xi’an, Shaanxi, 710061, China; ^2^ Department of Oncology, Second Affiliated Hospital of Xi’an Jiaotong University, Xi’an, Shaanxi, 710004, China

**Keywords:** interluekin-12, cancer risk, polymorphism, meta-analysis

## Abstract

Many molecular epidemiologic studies have explored the possible links between interleukin-12 (IL-12) polymorphisms and various cancers. However, results from these studies remain inconsistent. This meta-analysis is aimed to shed light on the associations between three common loci (rs568408, rs2243115, rs3212227) of IL-12 gene and overall cancer risk. Our meta-analysis finally included 33 studies comprising 10,587 cancer cases and 12,040 cancer-free controls. Odds ratios (ORs) and 95% confidence intervals (CIs) were used to assess the cancer risk. We observed a significant association between IL-12B rs3212227 and overall cancer risk, especially in hepatocellular carcinoma, nasopharyngeal cancer, and among Asians. IL-12A polymorphisms (rs2243115 and rs568408) were found no influence on overall cancer risk. Nevertheless, stratification analyses demonstrated that rs568408 polymorphism contributed to increasing cancer risk of Caucasians and cervical cancer. And, rs2243115 may enhance the risk of brain tumor. These findings provided evidence that IL-12 polymorphisms may play a potential role in cancer risk.

## INTRODUCTION

According to the data provided by *Cancer statistics, 2017*, around 1,688,780 new cancer patients and 600,920 cancer-related deaths will develop in America in 2017 [1]. And, it was estimated that a number of 4,292,000 new cancer patients and 2,814,000 cancer-related deaths would occur in China in 2015 [2]. These data declared that cancer has represented a substantial public health burden. It has been proved that cancer was a multifactorial disease due to dynamic interactions between the environmental exposures and host genetic background. The mechanisms of inherited factors effecting on tumor is still not known clearly, though the carcinogenesis and tumor-immunity function of cytokines have been well established [3]. In fact, one of the pivotal control mechanisms in pathogenesis of tumor was considered to be cytokine-mediated immunity [4]. For instance, interferon, a cytokine with strongly anti-tumor effects, was widely used in treatment of many cancers, especially for hematological malignant tumor such as chronic myeloid leukemia and hairy cell leukemia [5]. Likewise, Interleukins (ILs) were promising in immunotherapy for many cancers because they can enhance the immune response against both infections and tumors. In previous clinical trials several candidates of ILs (such as IL-1a, IL-2, and IL-18) were failed to show corresponding effectiveness on tumors, and other ILs (including IL-7, IL-10, IL-15 and IL-21) showed severe adverse side effects during treatment [6]. Thus, exploration and discovery new, effective immune-modulators are imperative. Interleukin-12 (IL-12), which is characteristic as a link between the innate and acquired immune response [7], is one of the most potential candidates for tumor immunotherapy.

IL-12 is mainly produced by activated antigen-presenting cells, such as macrophages, dendritic cells, and monocytes. As a heterodimeric cytokine, IL-12 protein is formed by two disulfide-linked polypeptide chains comprising p35 and p40, which are encoded by IL-12A gene and IL-12B gene, independently. And it mapped to human chromosomes 3p12-q13.2 and 5q31-33 [8]. IL-12 can potently induce the differentiation of naive CD4+ T cell to Th1 cells and augment cytotoxic T lymphocyte responses, as well as promote activation of natural killer (NK) cells, and secretion of interferon-γ [9]. Moreover, IL-12 can impair the mechanism of neoangiogenesis in tumors by antagonizing the pro-angiogenic signals. Animal experiments extensively showed that both nonspecific and specific antitumor immune responses were enhanced after transfecting IL-12 gene into tumor cells [10, 11]. In addition, clinical trials indicated that serum level of IL-12 was related to the severity of gastric cancer patients [12], and also influenced the progression of colorectal cancer [13].

Because of these pleiotropic activities of IL-12, dozens of molecular epidemiologic studies have explored the influences of IL-12 polymorphisms on susceptibility of various cancers, including hepatocellular carcinoma [14–19], colorectal cancer [13, 20, 21] and gastric cancer [22–24], etc. The most commonly fascinating loci were rs3212227 in IL-12B gene, rs568408 and rs2243115 in IL-12A genes, perhaps owing to their function to influence IL-12 gene expression, reduce protein synthesis, and subsequently result in cancer. Unfortunately, the functional role on cancer risk that IL-12 gene polymorphisms played was still uncertain. For instance, a study performed by Sun et al. found that IL-12A rs568408 may increase the susceptibility on colorectal cancer [20], being contrary to the conclusion of the study by Huang et al. which suggested IL-12A rs568408 was not associated with colorectal cancer[21]. And, a previous study reported that the loci of rs3212227 in IL-12B gene definitely contributed to increasing cervical cancer risk in Chinese population [25]. However, another study found IL-12B rs3212227 polymorphism exhibited a protective effect [26], while an earlier study showed that there was no relationship between cervical cancer and IL-12B rs3212227 in Korean population [27]. Hence, we conducted this study according to currently published data to investigate the precise relationship between these three common variants and various cancers risk.

## RESULTS

### Characteristics of eligible studies

**Figure 1:**
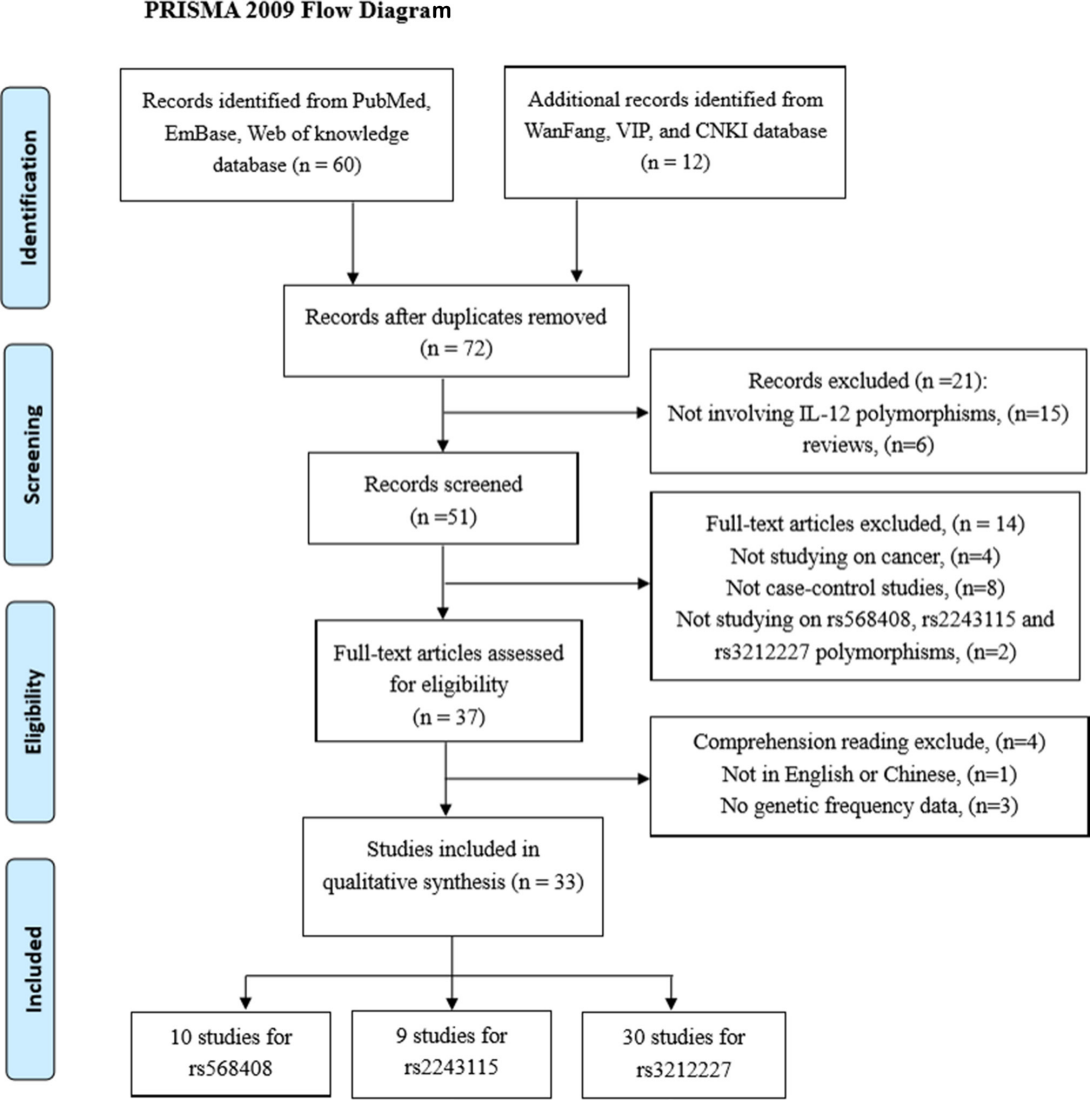
The flow diagram of the meta-analysis, according to the PRISMA 2009 CNKI: China National Knowledge Infrastructure.

As the flow diagram showed Figure 1, we identified a total of 72 publications from the PubMed, web of knowledge, Embase, WanFang, VIP and CNKI databases using the search words, fifteen obviously irrelevant studies and six reviews were excluded firstly. Fourteen studies were excluded from our meta-analysis after screening the titles and abstracts because they were not researching on rs568408, rs2243115 and rs3212227 polymorphisms (2 studies) or cancer (4 studies), and 8 studies were excluded for not case-control studies. The remaining 37 studies evaluated for eligibility by reading the full-text; 3 studies were excluded owing to either lack of complete data or presence of irrelevant data that focused on other IL-12 polymorphisms; one study was written neither in English nor in Chinese. Ultimately, 33 studies with 10,587 cancer cases and 12,040 cancer-free controls were elected in this meta-analysis to explored the relationship of IL-12A rs568408 (10 studies), IL-12A rs2243115 (9 studies), and IL-12B rs3212227 (30 studies) polymorphisms and cancer risk. According to the Newcastle-Ottawa Scale (NOS), all included studies obtained a score of 6 or more, which was defined as a high quality. Among these, 3 studies were written in Chinese and 30 studies in English. The genotype frequencies in 2 studies [17, 18] were not provided completely, we could only evaluated the data based on dominant model. The eligible studies involved several different cancer types including hepatocellular, colorectal, gastric, cervical, breast, prostate, osteosarcoma, esophageal, brain, cervical, ovarian, nasopharyngeal, lung, Non-Hodgkin lymphoma, and cutaneous malignant melanoma. Every study was in according with HWE except for two [20, 28]. The main characteristics of the included studies are presented in Table 1. The distributions of rs568408, rs2243115, and rs3212227 polymorphisms of IL-12 among cases and controls are shown in Table 2.

**Table 1:** Characteristics of the studies included in the meta-analysis

Study	Year	Country	Ethnic	Method	Source of control	Cancer type	Case/control	SNP.	NOS
Tan[14]	2015	China	Asian	iMLDR	Population	HCC	395/686	1,2,3	7
Sun[20]	2015	China	Asian	PCR-RFLP	Hospital	CRC	257/236	1,2,3	7
Yin[22]	2015	China	Asian	SNPscan	Hospital	GAC	234/476	2,3	7
Jafarzadeh[35]	2015	Iran	Asian	PCR-RFLP	Population	BC	100/100	3	6
Winchester[36]	2015	USA	Caucasian	MassArray	Population	PC	566/530	3	9
Saxena[15]	2014	India	Asian	PCR-RFLP	Population	HCC	59/153	3	6
wang[37]	2013	China	Asian	PCR-RFLP	Population	OSC	106/210	1,2,3	7
Sun[38]	2013	China	Asian	SNPscan	Hospital	EC	380/380	3	8
Jaiswal[39]	2013	Indian	Asian	PCR-RFLP	Hospital	BLC	200/200	3	6
Tao[40]	2012	Mixed	Asian	PCR-RFLP	Population	EC	426/432	1,3	6
Sima[41]	2012	China	Asian	PCR-RFLP	Hospital	BT	170/222	2,3	7
Kaarvatn[42]	2012	Croatia	Caucasian	TaqMan	Mixed	BC	191/194	3	6
Carvalho[26]	2012	Brazil	Mixed	PCR-RFLP	Population	CC	162/76	3	6
Roszak[25]	2012	Poland	Caucasian	PCR-RFLP	Population	CC	405/405	1,3	7
Hu[43]	2012	China	Asian	PCR-RFLP	Hospital	OC	92/38	3	6
Huang[21]	2012	China	Asian	PCR-RFLP	Hospital	CRC	410/450	3	6
Liu[16]	2011	China	Asian	PCR-RFLP	population	HCC	869/891	1,2,3	9
Chaaben[44]	2011	Tunisia	African	Taqman	population	NPC	247/284	3	6
Ter-Minassian[45]	2011	USA	Caucasian	MassArray	Population	BT	261/319	2	8
Yang[19]	2011	China	Asian	Taqman	Hospital	HCC	608/612	3	6
Wu[24]	2009	China	Asian	PCR-RFLP	Population	GC	1035/1073	3	7
Chen[46]	2009	China	Asian	PCR-RFLP	population	CC	404/404	1,2,3	9
Miteva[13]	2009	Bulgaria	Caucasian	PCR–RFLP	Hospital	CRC	85/134	3	6
Wei[47]	2009	China	Asian	PCR–RFLP	Hospital	NPC	302/310	3	6
Zhao[48]	2009	China	Asian	PCR–RFLP	Hospital	BT	210/220	3	6
Ognjanovic[17]	2009	USA	Caucasian	Taqman	population	HCC	117/223	3	9
Han[27]	2008	Korea	Asian	PCR–RFLP	Hospital	CC	150/179	3	7
Hou[23]	2007	USA	Caucasian	TaqMan	Population	GC	257/428	1	6
Lee[49]	2007	USA	Asian	TaqMan	Population	LC	119/113	1	8
Wang[50]	2006	USA	Caucasian	TaqMan	Population	NHL	1130/941	1,3	9
Nieters[18]	2005	China	Asian	PCR–RFLP	Hospital	HCC	250/250	3	8
Howell[51]	2003	UK	Caucasian	ARMS-PCR	Hospital	CMM	145/229	3	7

PCR: polymerase chain reaction; RFLP: restriction fragment length polymorphism; HCC: hepatocellular carcinoma; GC: gastric cancer; CRC: colorectal cancer; BC: breast cancer; PC: prostate cancer; OSC: osteosarcoma; EC: esophageal cancer; BLC: bladder cancer; BT: brain tumor; CC: cervical cancer; OC: ovarian carcinoma; NPC: nasopharyngeal cancer; GC: gastric cancer; LC: lung cancer; NHL: non-Hodgkin lymphoma; CMM: cutaneous malignant melanoma. SNP No.1: rs568408 (3′ UTR G>A) in IL-12A; 2: rs2243115 (5′UTR T>G) in IL-12A; 3: rs3212227 (3′ UTR A>C) in IL-12B; NOS: the Newcastle-Ottawa Scale.

**Table 2:** Genotype distribution and allele frequency of IL-12 polymorphisms

First author	Genotype (N)								Allele frequency (N)				MAF	HWE
	Case				Control				Case		Control			
	Total	AA	AB	BB	Total	AA	AB	BB	A	B	A	B		
**rs568408**														
Tan 2015[14]	395	313	76	6	686	511	161	14	702	80	1183	189	0.102	0.750
Sun 2015[20]	257	172	83	2	236	181	55	0	427	89	417	55	0.172	0.040
Wang2013[37]	106	66	37	3	210	156	47	7	169	61	359	61	0.265	0.150
Tao 2012[40]	430	276	142	12	432	304	112	16	694	184	720	144	0.210	0.170
Roszak 2012[25]	405	253	128	24	450	306	125	19	634	140	737	163	0.181	0.180
Liu 2011[16]	802	504	277	21	861	631	220	10	1285	289	1482	240	0.184	0.060
Chen 2009[46]	404	253	145	6	404	285	112	7	651	221	682	126	0.253	0.290
Hou 2007[23]	257	211	8	38	428	306	112	10	430	8	724	132	0.018	0.950
Lee 2007[49]	118	101	17	0	113	81	31	1	219	81	193	33	0.270	0.290
Wang 2006[50]	941	671	238	32	1130	839	270	21	1580	238	1948	312	0.131	0.890
**rs2243115**														
Tan 2015[14]	395	326	63	6	685	549	128	8	715	75	1226	144	0.095	0.861
Sun 2015[20]	257	225	31	1	236	212	24	0	481	33	448	24	0.064	0.411
Yin 2015[22]	235	197	36	2	466	387	75	4	430	40	849	83	0.085	0.862
Wang2013[37]	106	89	16	1	210	175	31	4	194	18	381	39	0.085	0.073
Sun 2013[38]	368	311	56	1	370	308	58	4	678	58	674	66	0.079	0.499
Sima 2012[41]	170	140	25	5	222	198	24	0	305	35	420	24	0.103	0.395
Liu 2011[16]	861	735	125	1	874	732	137	5	1595	127	1601	147	0.074	0.604
Ter-Minassian 2011[45]	248	115	113	20	317	180	114	23	343	153	474	160	0.308	0.403
Chen 2009[46]	404	342	60	2	404	337	64	3	744	64	738	70	0.079	0.984
**rs3212227**														
Tan 2015[14]	395	104	201	90	686	200	347	139	409	381	747	625	0.482	0.605
Sun 2015[20]	257	69	140	48	236	71	124	41	278	236	266	206	0.459	0.295
Yin 2015[22]	234	60	132	42	466	152	221	93	252	216	525	407	0.462	0.436
Jafarzadeh 2015[35]	100	58	32	10	100	59	33	8	148	52	151	49	0.260	0.280
Winchester 2015[36]	866	535	274	57	830	568	227	35	1344	388	1363	297	0.224	0.046
Saxena 2014[15]	59	19	31	9	148	63	71	14	69	49	197	99	0.415	0.345
Wang 2013[37]	106	27	50	29	210	78	101	31	104	108	257	163	0.509	0.585
Sun 2013[38]	368	116	176	76	370	112	179	79	408	328	403	337	0.446	0.635
Jaiswal 2013[39]	200	87	94	19	200	111	74	15	268	132	296	104	0.330	0.590
Tao 2012[40]	426	109	216	101	432	148	213	71	434	418	509	355	0.491	0.701
Sima 2012[41]	170	66	86	18	222	71	116	35	218	122	258	186	0.359	0.275
Kaarvatn 2012[42]	191	126	59	6	194	104	73	17	311	71	281	107	0.186	0.419
Carvalho 2012[26]	162	100	49	13	76	31	37	8	249	75	99	53	0.231	0.531
Roszak 2012[25]	405	212	174	19	450	289	151	10	598	212	729	171	0.262	0.056
Hu 2012[43]	92	16	42	34	38	13	16	9	74	110	42	34	0.598	0.360
Huang 2012[21]	410	93	219	98	450	124	230	96	405	415	478	422	0.506	0.578
Liu 2011[16]	831	249	422	160	844	272	414	158	920	742	958	730	0.446	0.983
Chaaben 2011[44]	247	74	124	49	284	135	118	31	272	222	388	180	0.449	0.497
Yang2011[19]	608	156	309	143	612	195	302	115	621	595	692	532	0.489	0.919
Wu 2009[24]	1035	347	508	180	1073	333	554	186	1202	868	1220	926	0.419	0.086
Chen 2009[46]	404	127	199	78	404	150	185	69	453	355	485	323	0.439	0.357
Miteva 2009[13]	85	50	31	4	134	80	45	9	131	39	205	63	0.229	0.443
Wei 2009[47]	302	58	165	79	310	98	152	60	281	323	348	272	0.535	0.938
Zhao 2009[48]	210	38	115	57	220	70	106	44	191	229	246	194	0.545	0.736
Ognjanovic 2009[17]	117	57	/	/	223	128	/	/	/	/	/	/	/	/
Tamandani 2009[28]	200	63	134	3	200	88	104	8	260	140	280	120	0.350	0.001
Han 2008[27]	150	32	87	31	179	52	88	39	151	149	192	166	0.497	0.877
Wang 2006[50]	926	565	310	51	1110	641	397	72	1440	412	1679	541	0.222	0.322
Nieters 2005[18]	249	56	/	/	250	72	/	/	/	/	/	/	/	/
Howell2003[51]	145	95	42	8	229	139	77	13	232	58	355	103	0.200	0.591

A: the major allele, B: the minor allele, MAF: minor allele frequencies; HWE: Hardy–Weinberg equilibrium.

### Quantitative synthesis of rs568408 and rs2243115 polymorphisms

In the meta-analysis, we derived data from 10 studies including 4115 cancer cases and 4950 cancer-free controls for rs568408, 9 studies containing 3004 cancer cases and 3784 cancer-free controls for rs2243115, respectively.

**Figure 2:**
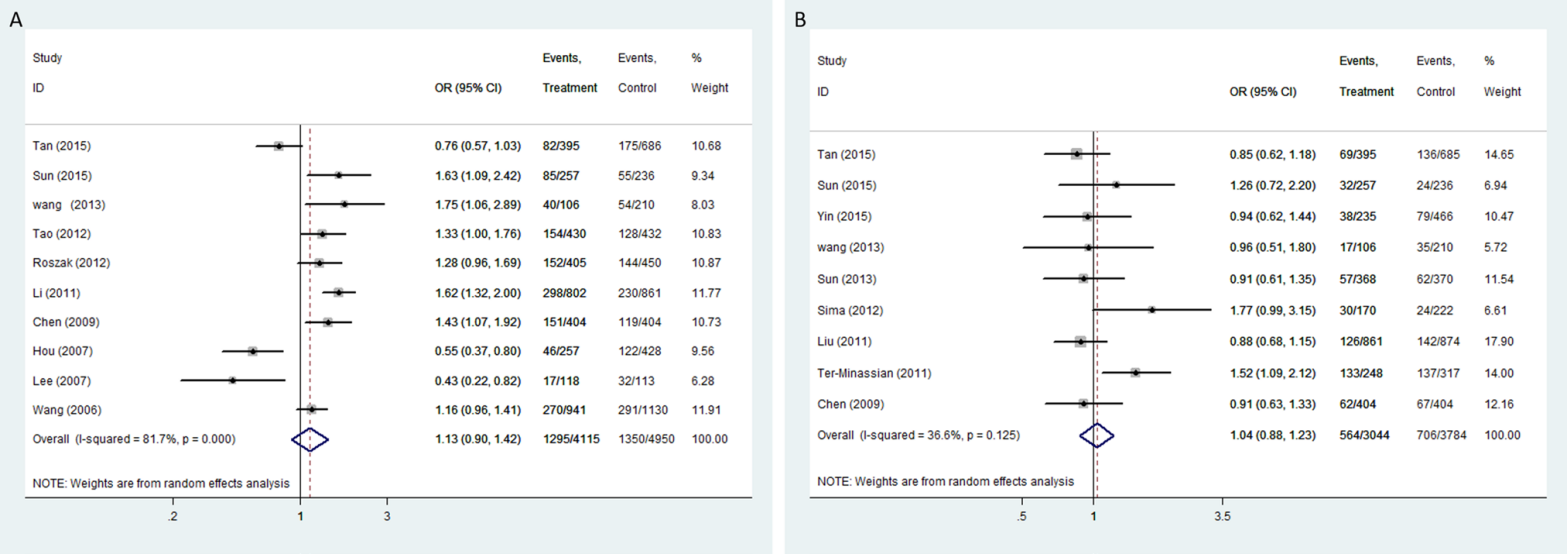
Forest plot of OR with 95%CI for the IL-12 polymorphisms with cancer risk under dominant model (**A**) rs568408; (**B**) rs2243115). CI: confidence interval, OR: odds ratio.

The pooled analysis proved that IL-12A rs568408 polymorphisms was associated with overall cancer risk on allele comparison (A vs. G: OR = 1.18, 95%CI: 1.01-1.38, *P* = 0.04). However, no significant association was found after excluding one study [20] not according to the HWE (All *P* > 0.05, Figure 2). In stratified analysis by ethnicity, we observed an increased risk of overall cancers among Caucasians (recessive model: OR = 2.63, 95% CI = 1.05-6.56; homozygote model: OR = 2.46, 5% CI = 1.20-5.06; allele model: OR = 1.19, 95% CI = 1.05-1.35). The subgroup analysis by cancer type showed rs568408 polymorphism increased cervical cancer risk under three genetic models (allele model: OR = 1.28, 95% CI = 1.07-1.52; heterozygous model: OR = 1.34, 95% CI = 1.09-1.66; dominate model: OR = 1.35, 95% CI = 1.10-1.65). Rs2243115 polymorphism was associated with brain tumor under dominate model (OR = 1.58, 95% CI = 1.18-2.11), heterozygous model (OR = 1.53, 95 % CI = 1.13-2.07) and allele model (OR = 1.52, 95% CI = 1.03-2.24). However, the subgroup analysis based on source of controls proved IL-12A polymorphisms (rs568408 and rs2243115) have no influence on cancer susceptibility in both hospital-based and population-based controls subgroups (all *P* > 0.05).

### Quantitative synthesis of rs3212227 polymorphism

We gained 9950 patients and 11180 control subjects from 30 studies of rs3212227 polymorphism. IL-12B rs3212227 was proved to enhance overall cancer risk (C vs. A: OR = 1.15, 95%CI: 1.05-1.25; CC vs. AA: OR = 1.32, 95%CI: 1.11-1.56; AC vs. AA: OR= 1.21, 95%CI: 1.08-1.35, *P* = 0.001; AC+CC vs. AA: OR = 1.24, 95%CI: 1.10-1.40, *P* < 0.001; CC vs. AC+AA: OR = 1.17, 95%CI: 1.04-1.31). The similar results were obtained after excluding studies with HWE disequilibrium (C vs. A: OR = 1.14, 95%CI: 1.05-1.25; CC vs. AA: OR = 1.33, 95%CI: 1.12-1.58; AC vs. AA: OR = 1.19, 95%CI: 1.06-1.34; AC+CC vs. AA: OR = 1.23, 95%CI: 1.09-1.38; CC vs. AC+AA: OR = 1.18, 95%CI: 1.05-1.32).

**Figure 3:**
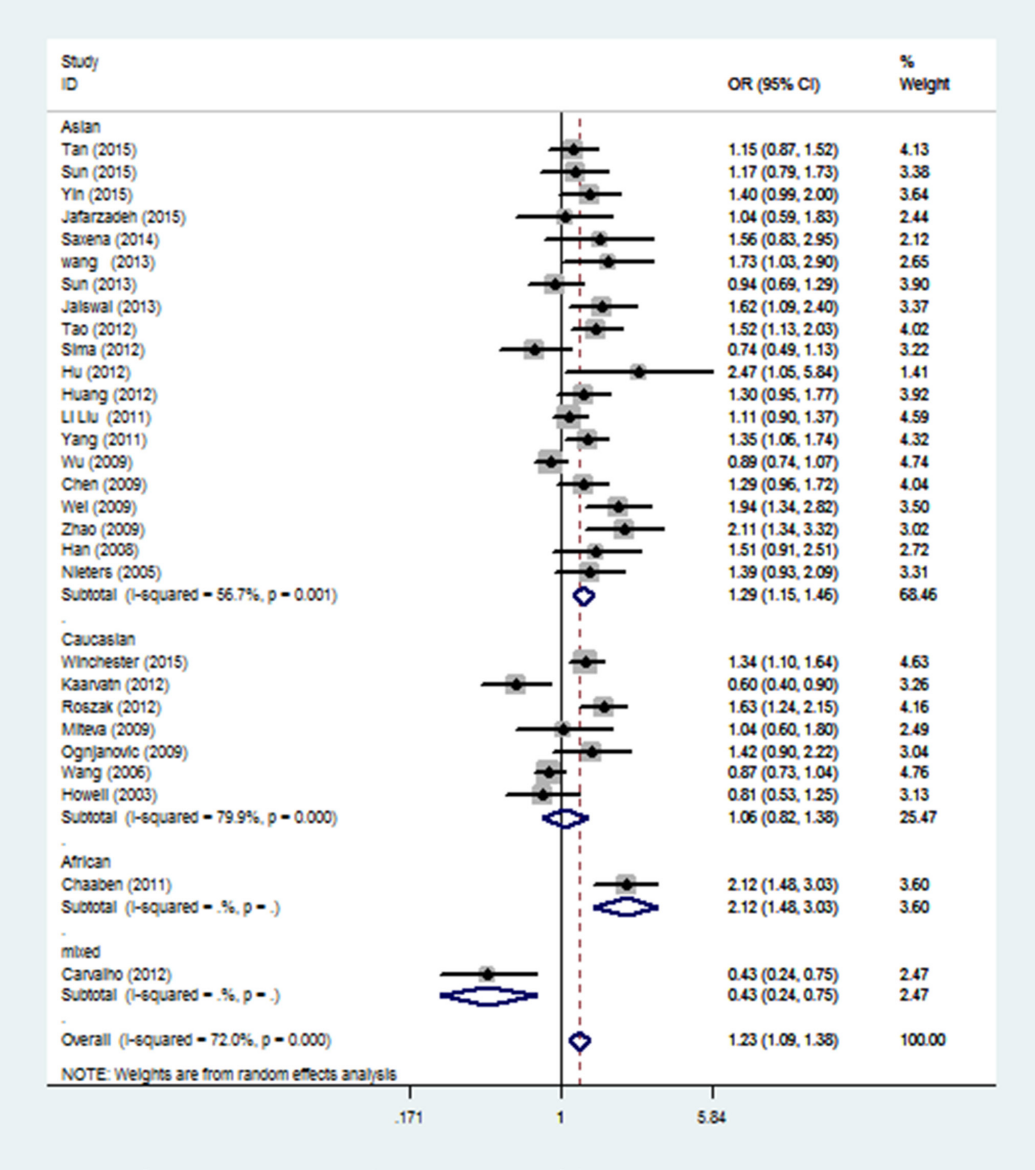
Stratified analysis by ethnicity for the association between IL-12 rs3212227 polymorphism and cancer risk under dominant model (CC + AC vs. AA) according to the HWE CI: confidence interval, OR: odds ratio, HWE: Hardy–Weinberg equilibrium.

**Figure 4:**
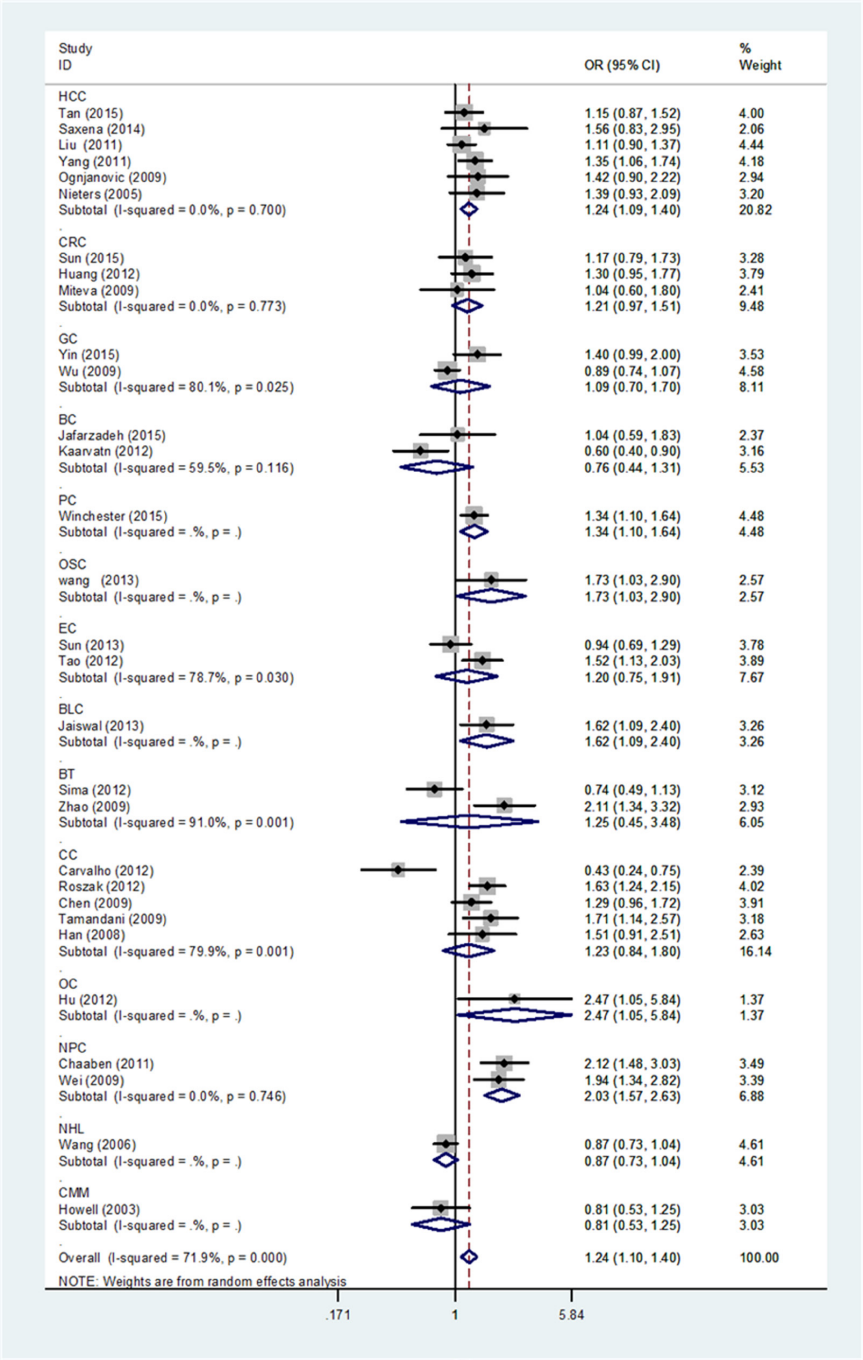
Stratified analysis by cancer type for the association between IL-12B rs3212227 polymorphism and cancer risk under dominant model (CC + AC vs. AA) according to the HWE CI: confidence interval, OR: odds ratio. HCC: hepatocellular carcinoma; GC: gastric cancer; CRC: colorectal cancer; BC: breast cancer; PC: prostate cancer; OSC: osteosarcoma; EC: esophageal cancer; BLC: bladder cancer; BT: brain tumor; CC: cervical cancer; OC: ovarian carcinoma; NPC: nasopharyngeal cancer; GC: gastric cancer; LC: lung cancer; NHL: non-Hodgkin lymphoma; CMM: cutaneous malignant melanoma, HWE: Hardy–Weinberg equilibrium.

When stratification analysis performed by ethnicity (Figure 3), rs3212227 polymorphism exhibited to increase overall cancer risk among Asians (AC+CC vs. AA: OR = 1.29, 95%CI: 1.15-1.46; CC vs. AC+AA: OR = 1.17, 95%CI: 1.06-1.30; CC vs. AA: OR = 1.38, 95%CI: 1.17-1.62; AC vs. AA: OR = 1.25, 95%CI: 1.11-1.40; C vs. A: OR = 1.18, 95%CI: 1.09-1.28). In subgroup analysis by cancer type (Figure 4), rs3212227 was found to increase the risk of hepatocellular carcinoma (AC+CC vs. AA: OR = 1.24, 95%CI: 1.09-1.40; CC vs. AC+AA: OR = 1.17, 95%CI: 1.00-1.37; CC vs. AA: OR = 1.30, 95%CI: 1.07-1.59; AC vs. AA: OR = 1.17, 95%CI: 1.02-1.35; C vs. A: OR = 1.14, 95%CI: 1.04-1.25) and nasopharyngeal carcinoma (AC+CC vs. AA: OR = 2.03, 95%CI: 1.57-2.63; CC vs. AC+AA: OR = 1.66, 95%CI: 1.23-2.25; CC vs. AA: OR = 2.49, 95%CI: 1.75-3.54; AC vs. AA: OR = 1.88, 95%CI: 1.43-2.47; C vs. A: OR = 1.60, 95%CI: 1.34-1.90). When conducted a stratified analysis by the source of controls, rs3212227 polymorphism displayed an increased cancer risk among population-based studies and hospital-based studies in different comparison models. The detail results were listed in Supplementary Table 1.

### Heterogeneity and sensitivity analysis

**Figure 5:**
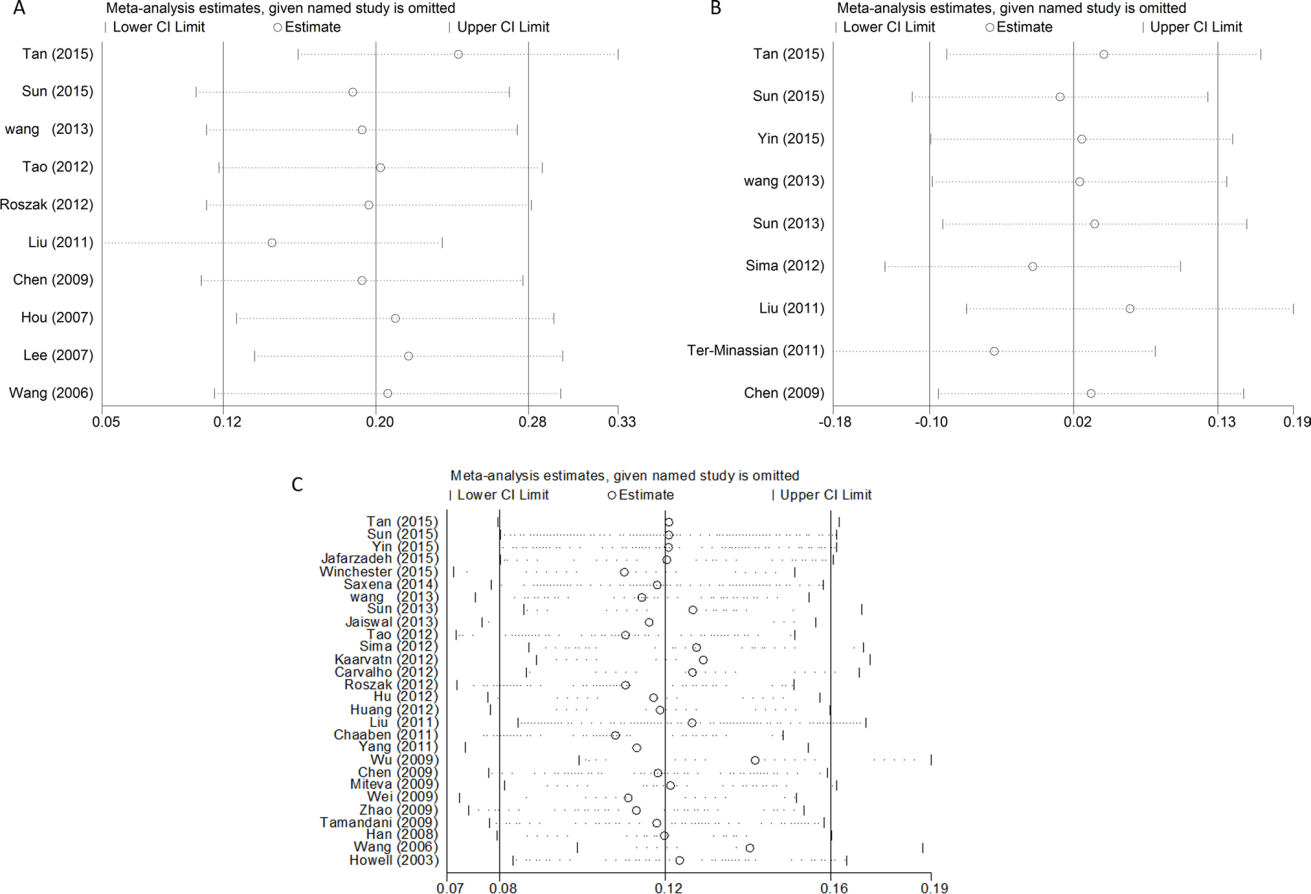
Sensitivity analysis of the associations between IL12 polymorphisms and cancer risk (**A**) rs568408; (**B**) rs2243115; (**C**) rs3212227).

For the overall study, statistically significant heterogeneity existed in rs568408 polymorphism (AA+GA vs. GG, *P* < 0.0001, I^2^ = 82%; AA vs. GA+GG, *P* = 0.001, I^2^ = 68%; AA vs. GG, *P* = 0.009, I^2^ = 59%; GA vs. GG, *P* < 0.0001, I^2^ = 88%; A vs. G, *P* < 0.0001, I^2^ = 70%) and rs3212227 polymorphism (AC+CC vs. AA, *P* < 0.0001, I^2^ = 72%; CC vs. AA, *P* < 0.0001, I^2^ = 65%; AC vs. AA, *P* < 0.0001, I^2^ = 66%; C vs. A, *P* < 0.0001, I^2^ = 74%). Therefore, we conducted further subgroup analyses by cancer type, ethnicity and source of control (Supplementary Table 1). The sequential leave-one-out sensitivity analysis was adopted to evaluate each individual study's influence on the overall risk estimates. However, the results proved there was no single study could skew the pooled ORs significantly, indicating the credibility and reliability of this meta-analysis (shown in Figure 5).

### Publication bias

**Figure 6:**
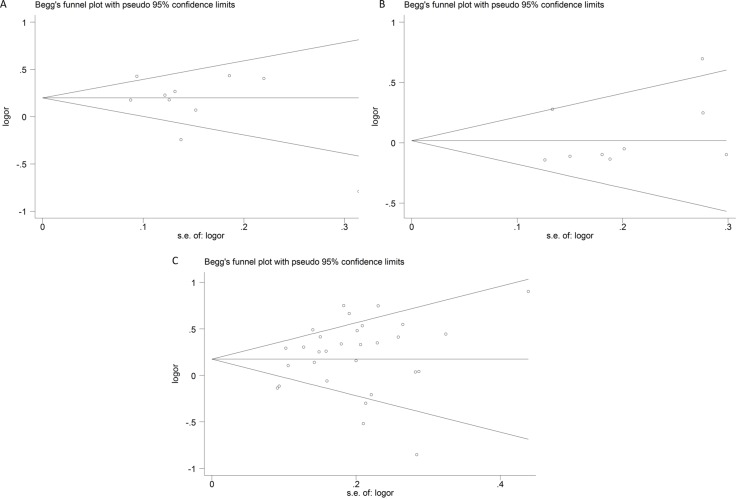
Funnel plots of publication bias (**A**) rs568408; (**B**) rs2243115; (**C**) rs3212227).

Publication bias of the eligible studies of these IL-12 polymorphisms was accessed by funnel plot and Egger's test. The symmetrical shapes of the funnel plots (Figure 6) and further statistical evidence provided by Egger's test (Table 3) showed the absence of publication bias (*P* > 0.05).

**Table 3:** Egger's test for publication bias test of IL-12 polymorphisms

Egger’s test		Coefficient	SE	t	P > |t|	95%CI
rs568408	slope	0.552139	0.228511	2.42	0.046	0.0117964–1.092481
	bias	−3.03665	1.803099	−1.68	0.136	−7.300299–1.227003
rs2243115	slope	−0.20484	0.284498	−0.72	0.495	−0.877572–0.467887
	bias	1.303655	1.599617	0.81	0.442	−2.837692–6.454866
rs3212227	slope	−0.0187827	0.1555829	−0.12	0.905	−3380125–0.300447
	bias	1.225427	0.9578139	1.28	0.212	−7398447–3.190699

SE: standard error; 95%CI: 95% confidence interval.

## DISCUSSION

Attracted to the effects of IL-12 polymorphisms in the onset risk of cancer, many researchers performed numerous molecular epidemiological studies to explore the connection. Regrettably, they were failed to achieve a coincident conclusion. Existing epidemiological studies showed a higher interest on the SNP of rs3212227 in IL-12B gene, but put less focus on the rs568408 and rs2243115 SNPs of IL-12A genes. Only one meta-analysis (including eighteen studies) focused on IL-12A polymorphisms and suggested that both rs568408 and rs2243115 polymorphisms of IL-12A did not connect with cancer susceptibility [29]. In our meta-analysis, we observed that rs568408 polymorphism increased cervical cancer risk and overall cancer risk among Caucasians, while rs2243115 polymorphism may be a high risk factor of brain tumor. And, the mutant C allele of rs3212227 polymorphism was proved to contribute to overall cancer's development, especially for hepatocellular carcinoma, nasopharyngeal cancer and among Asians.

What we found in this meta-analysis is contrary to the previous meta-analyses [29, 30], our study proved no association between cervical cancer and rs3212227 polymorphism, while rs568408 and rs2243115 polymorphisms were associated with cervical cancer and brain tumor respectively. Compared to the previous meta-analyses, this study included more eligible studies, conducted more detailed subgroup analyses, and also performed stringent quality control during data statistics. Thus, our results are more comprehensive and persuasive. In the past years, we considered only IL-12B polymorphism played an important role in cancer development and looked down on the function of IL-12A. This meta-analysis indicated that we should perform more comprehensive investigation both on the respective function and interaction of IL-12A and IL-12B.

As is depicted in introduction, IL-12 is a multiple effective cytokine that links the innate (NK cells) and acquirs (cytotoxic T lymphocytes) immune response [7]. Structurally, IL-12 is a heterodimer consisted of a 35 kD α-chain (p35 subunit) and a 40 kD β-chain (p40 subunit), the latter was shared with IL-23, another important member from IL-12 family. P35 and p40 are encoded by IL-12 A and IL-12 B gene, respectively. And, IL-12 p40 is produced in excess over the IL-12 and IL-23 heterodimers in average level [31]. We guess the amount of activated IL-12B gene was much more than activated IL-12A gene, thus we observed more mutation frequency in IL-12B rs3212227. In fact, both IL-12A and IL-12B gene have played a role in the development of cancer. Therefore, we speculated IL-12 polymorphisms may alter the IL-12 gene expression, decrease functional IL-12 protein synthesis, which ultimately brought about immune system dysfunction and lead to the development of malignant tumors. The clinical trials of IL-12 were rather disappointing, because systemic administration in cancer treatment was observed defined anti-tumor efficacy and severe toxic effects. Thus, it was now vigorously promoted to develop the gene therapy vectors which can express activated IL-12 locally in tumors. New avenues for IL-12 in cancer have been opened with technical advances in recent years, which are currently under research [32]. Our meta-analysis provided a direction to the further vivo and *in vitro* experiments.

Some possible limitations in this study need to be attended. Firstly, some potential source of heterogeneity, such as sex and environmental exposures, may have influence on the final results of the present study. Secondly, environmental factors were failed to evaluate, because lack of relevant data, such as family history, age, lifestyle, which might alter the real relation of IL-12 polymorphisms and the cancer risk. Thirdly, studies written in a third language were excluded (not in English or Chinese), thus, selection bias is inevitable. Fourthly, although we pooled all eligible publications together in our study, the sample size for some types of cancer were still small. For instance, there was only one or two literature available for cervical cancer. Thus, we should be cautious to explain our results. Finally, publication bias may exist in our meta-analysis because we could not gain the unpublished data.

In conclusion, this meta-analysis draws a reliable conclusion that these IL-12 polymorphisms (rs568408, rs2243115 and rs3212227) might be high risk factors for cancer, which indicated IL-12 may serve as a promising marker for cancer screening. Further well-designed multi-center studies with larger sample sizes (especially for rs568408 and rs2243115 polymorphisms of IL-12A) and detailed gene-environmental interactions are warranted to confirm our findings.

## MATERIALS AND METHODS

### We followed the PRISMA statement to guide the process of this meta-analysis [33]

#### Search strategy

We obtained eligible studies prior to October 2016 from the PubMed, web of knowledge, Embase, WanFang, VIP and CNKI databases, using the terms: “cancer or carcinoma or tumor or neoplasm” and “polymorphism or variant or variation” and “interleukin-12 or IL-12”. In addition, we manually screened the references in the relevant reviews to ensure all relevant studies were not missed. Studies elected in this meta-analysis met the criteria as follows: (1) case-control studies evaluated the association of IL-12A rs568408, IL-12A rs2243115, or IL-12B rs3212227 polymorphisms with various cancer risks; (2) genotype distributions were sufficient for both cases and controls for data extraction; (3) published in English or Chinese. And we adopted following exclusion criteria: (1) repeat of previous studies, reviews, or abstracts; (2) not case-control design; (3) absence of detailed genotyping data; (4) duplication of previous data. If the overlap of data appeared in different publications, the paper included more samples were chosen.

### Data extraction and quality assessment

Two investigators independently elected for following information from the all eligible studies: first author, publication year, cancer type, country, ethnicity, source of controls, genotyping platform, total number of cases and controls, genetic distribution among cases and controls, and Hardy–Weinberg equilibrium (HWE) *P value* of controls. The subgroup analyses were performed according to cancer type, ethnicity (Asians, Caucasians), and source of controls. All patients in case group were confirmed by histology or pathology. The Newcastle-Ottawa Scale (NOS) was used to assess the quality of the eligible studies [34], The total scores ranged from 0 to 10, with higher scores indicating better quality. Score of 0–5, 6-9 was considered as indicative of a low-, high-quality study, respectively. To resolve all disagreements of two investigators, a discussion with a senior investigator was conducted until a consensus was reached.

### Statistical analysis

To evaluate the cancer risk associated IL-12 polymorphisms (rs568408, rs2243115, rs3212227), we calculated ORs and 95% CI based on the genotypes in cases and controls. Five different genetic models were evaluated: (i) BB+ AB vs. AA (dominant model), (ii) BB vs. AA (homozygote model), (iii) AB vs. AA (heterozygote model), (iv) BB vs. AA+ AB (recessive model), (v) B vs. A (allele comparison). “A” represents the wild allele, while “B” represents the mutation allele. Heterogeneity among the studies was checked by Chi-square-based Q statistic and I^2^ test, and it was regarded as homogeneous if *P* > 0.10 in Chi-square-based Q statistic. When the effects were assumed to be homogeneous, we used the fixed-effects model to assess the pooled OR. Otherwise, the random-effects model was chosen. Furthermore, source of heterogeneity among studies was explored by subgroup analyses base on cancer type, ethnicity and source of control. We adopted Begg's and Egger's test to evaluate the possible sources of bias. Sensitivity analysis was chiefly performed by orderly removing individual study and rechecked the pooled ORs to assess the stability of the final results. All statistical analyses in this study were performed by software STATA (Version 14.0; Stata Corp, College Station, TX). A statistical significance was considered if *P* < 0.05 and all the statistics were two-sided.

## SUPPLEMENTARY MATERIALS FIGURES AND TABLES




