# Supplementary material for: Role of interleukin-12 gene polymorphisms in the onset risk of cancer: a meta-analysis
Source: Oncotarget. 2017 Mar 10;8(18):29795–807. doi: 10.18632/oncotarget.16080 (PMC5444704; doi:10.18632/oncotarget.16080)
Supplement: Supplementary file 1 [file oncotarget-08-29795-s001.pdf]

Role of interleukin-12 gene polymorphisms in the onset risk of cancer: a meta-analysis

Supplementary Materials

Supplementary Table 1: Meta-analysis of the associations between IL-12 polymorphisms and cancer risk. See Supplementary_Table_1
