# Supplementary material for: Role of interleukin-12 gene polymorphisms in the onset risk of cancer: a meta-analysis
Source: Oncotarget. 2017 Mar 10;8(18):29795–807. doi: 10.18632/oncotarget.16080 (PMC5444704; doi:10.18632/oncotarget.16080)
Supplement: Supplementary file 2 [file oncotarget-08-29795-s002.docx]

Supplementary Table 1: Meta-analysis of the associations between IL-12 polymorphisms and cancer risk.

| Comparisons | OR | 95%CI | P value | Heterogeneity | | Effects model |
| --- | --- | --- | --- | --- | --- | --- |
|  |  |  |  | I2 | P value |  |
| rs568408 |  |  |  |  |  |  |
| **B** vs. **A** | **1.18** | **1.01-1.38** | **0.040** | 70% | < 0.001 | R |
| HWE | 1.15 | 0.97-1.36 | 1.101 | 72% | < 0.001 | R |
| Asian | 1.10 | 0.84-1.46 | 0.490 | 82% | < 0.001 | R |
| Caucasian | **1.19** | **1.05-1.35** | **0.008** | 0% | 0.716 | F |
| HCC | 1.11 | 0.57-2.53 | 0.770 | 94% | < 0.001 | R |
| CC | **1.28** | **1.07-1.52** | **0.006** | 0% | 0.827 | F |
| **BB** vs. **AA** | 1.58 | 0.99-2.52 | 0.054 | 59% | 0.009 | R |
| HWE | 1.54 | 0.95-2.48 | 0.078 | 63% | 0.006 | R |
| Asian | 1.10 | 0.65-1.85 | 0.725 | 30% | 0.208 | F |
| Caucasian | **2.46** | **1.20-5.06** | **0.014** | 74% | 0.202 | R |
| HCC | 1.40 | 0.38-5.13 | 0.607 | 78% | 0.035 | R |
| CC | 1.37 | 0.79-2.35 | 0.260 | 0% | < 0.001 | F |
| **AB vs. AA** | 1.02 | 0.75-1.37 | 0.913 | 88% | < 0.001 | R |
| HWE | 0.96 | 0.70-1.33 | 0.821 | 89% | < 0.001 | R |
| Asian | 1.18 | 0.86-1.63 | 0.309 | 82% | < 0.001 | R |
| Caucasian | 0.58 | 0.24-1.40 | 0.222 | 95% | < 0.001 | R |
| HCC | 1.11 | 0.55-2.24 | 0.766 | 93% | < 0.001 | R |
| CC | **1.34** | **1.09-1.66** | **0.006** | 0% | 0.448 | F |
| **BB+AB** vs. **AA** | 1.13 | 0.90-1.42 | 0.302 | 82% | < 0.001 | R |
| HWE | 1.09 | 0.85-1.39 | 0.511 | 83% | < 0.001 | R |
| Asian | 1.16 | 0.84-1.60 | 0.379 | 83% | < 0.001 | R |
| Caucasian | 0.96 | 0.63-1.47 | 0.838 | 86% | 0.001 | R |
| HCC | 1.12 | 0.54-2.35 | 0.758 | 94% | < 0.001 | R |
| CC | **1.35** | **1.10-1.65** | **0.004** | 0% | 0.587 | F |
| **BB** vs. **AB+AA** | 1.51 | 0.89-2.57 | 0.123 | 68% | 0.001 | R |
| HWE | 1.47 | 0.85-2.52 | 0.166 | 71% | 0.001 | R |
| Asian | 1.02 | 0.64-1.63 | 0.923 | 18% | 0.298 | F |
| Caucasian | **2.63** | **1.05-6.56** | **0.039** | 84% | 0.002 | R |
| HCC | 1.36 | 0.45-4.09 | 0.589 | 69% | 0.072 | R |
| CC | 1.26 | 0.74-2.16 | 0.395 | 0% | 0.425 | F |
| rs2243115 |  |  |  |  |  |  |
| **B** vs. **A** | 1.03 | 0.88-1.21 | 0.712 | 44% | 0.074 | F |
| Asian | 0.97 | 0.83-1.14 | 0.733 | 27% | 0.217 | F |
| HCC | 0.88 | 0.73-1.06 | 0.177 | 0% | 0.881 | F |
| BT | **1.52** | **1.03-2.24** | **0.034** | 47% | 0.172 | F |
| **BB**vs**AA** | 1.04 | 0.61-1.78 | 0.888 | 12% | 0.337 | F |
| Asian | 0.86 | 0.41-1.80 | 0.692 | 15% | 0.310 | F |
| HCC | 0.64 | 0.11-3.75 | 0.618 | 57% | 0.126 | R |
| BT | 0.34 | 0.30-30.89 | 0.342 | 10% | 0.632 | F |
| **AB**vs**AA** | 1.03 | 0.88-1.20 | 0.719 | 21% | 0.260 | F |
| Asian | 0.95 | 0.83-1.10 | 0.489 | 0% | 0.823 | F |
| HCC | 0.88 | 0.71-1.08 | 0.212 | 0% | 0.670 | F |
| BT | **1.53** | **1.13-2.07** | **0.006** | 0% | 0.884 | F |
| **BB+AB** vs. **AA** | 1.04 | 0.88-1.23 | 0.671 | 37% | 0.125 | F |
| Asian | 0.95 | 0.83-1.09 | 0.478 | 0% | 0.519 | F |
| HCC | 0.87 | 0.71-1.07 | 0.184 | 0% | 0.873 | F |
| BT | **1.58** | **1.18-2.11** | **0.002** | 0% | 0.657 | F |
| **BB** vs. **AB+AA** | 0.99 | 0.61-1.61 | 0.980 | 4% | 0.401 | F |
| Asian | 0.87 | 0.42-1.81 | 0.714 | 14% | 0.320 | F |
| HCC | 0.65 | 0.11-3.91 | 0.638 | 58% | 0.122 | R |
| BT | 2.77 | 0.23-33.10 | 0.420 | 67% | 0.080 | R |
| rs3212227 |  |  |  |  |  |  |
| **B** vs. **A** | **1.15** | **1.05-1.25** | **0.002** | 74% | < 0.001 | R |
| HWE | **1.14** | **1.05-1.25** | **0.003** | 75% | < 0.001 | R |
| Asian | **1.18** | **1.09-1.28** | **0.000** | 59% | 0.001 | R |
| Caucasian | 1.00 | 0.77-1.30 | 0.998 | 85% | < 0.001 | R |
| HCC | **1.14** | **1.04-1.25** | **0.006** | 10% | 0.001 | F |
| CRC | 1.12 | 0.97-1.29 | 0.125 | 0% | 0.758 | F |
| BC | 0.79 | 0.44-1.41 | 0.425 | 76% | 0.040 | R |
| EC | 1.16 | 0.81-1.65 | 0.427 | 84% | 0.011 | R |
| CC | 1.08 | 0.78-1.48 | 0.651 | 82% | 0.083 | R |
| NPC | **1.60** | **1.34-1.90** | **0.000** | 8% | 0.297 | F |
| BT | 1.09 | 0.56-2.10 | 0.800 | 91% | 0.001 | R |
| GC | 1.00 | 0.87-1.14 | 0.943 | 25% | 0.247 | F |
| **BB** vs. **AA** | **1.32** | **1.11-1.56** | **0.001** | 65% | < 0.001 | R |
| HWE | **1.33** | **1.12-1.58** | **0.001** | 65% | < 0.001 | R |
| Asian | **1.38** | **1.17-1.62** | **0.000** | 56% | 0.002 | R |
| Caucasian | 1.00 | 0.58-1.73 | 1.000 | 74% | 0.002 | R |
| HCC | **1.30** | **1.07-1.59** | **0.009** | 14% | 0.323 | F |
| CRC | 1.26 | 0.93-1.70 | 0.141 | 0% | 0.604 | F |
| BC | 0.61 | 0.14-2.57 | 0.496 | 77% | 0.037 | R |
| EC | 1.34 | 0.66-2.75 | 0.421 | 85% | 0.011 | R |
| CC | 1.30 | 0.79-2.12 | 0.302 | 55% | 0.084 | R |
| NPC | **2.49** | **1.75-3.54** | **0.000** | 0% | 0.472 | F |
| BT | 1.16 | 0.28-4.87 | 0.837 | 91% | 0.001 | R |
| GC | 0.97 | 0.78-1.22 | 0.813 | 0% | 0.445 | F |
| **AB** vs. **AA** | **1.21** | **1.08-1.35** | **0.001** | 66% | < 0.001 | R |
| HWE | **1.19** | **1.06-1.34** | **0.003** | 65% | < 0.001 | R |
| Asian | **1.25** | **1.11-1.40** | **0.000** | 46% | 0.015 | F |
| Caucasian | 1.03 | 0.80-1.33 | 0.805 | 75% | 0.001 | R |
| HCC | **1.17** | **1.02-1.35** | **0.028** | 0% | 0.769 | F |
| CRC | 1.21 | 0.95-1.52 | 0.117 | 0% | 0.896 | F |
| BC | 0.76 | 0.53-1.10 | 0.146 | 6% | 0.303 | F |
| EC | 1.15 | 0.80-1.65 | 0.455 | 61% | 0.110 | R |
| CC | 1.12 | 0.70-1.80 | 0.643 | 83% | 0.001 | R |
| NPC | **1.88** | **1.43-2.47** | **0.000** | 0% | 0.874 | F |
| BT | 1.26 | 0.51-3.09 | 0.620 | 87% | 0.005 | R |
| GC | 1.13 | 0.66-1.91 | 0.658 | 85% | 0.011 | R |
| **BB+AB** vs. **AA** | **1.24** | **1.10-1.40** | **0.000** | 72% | < 0.001 | R |
| HWE | **1.23** | **1.09-1.38** | **0.001** | 72% | < 0.001 | R |
| Asian | **1.29** | **1.15-1.46** | **0.000** | 57% | 0.001 | R |
| Caucasian | 1.06 | 0.82-1.38 | 0.660 | 80% | < 0.001 | R |
| HCC | **1.24** | **1.09-1.40** | **0.001** | 0% | 0.700 | F |
| CRC | 1.21 | 0.969-1.51 | 0.093 | 0% | 0.773 | F |
| BC | 0.76 | 0.44-1.31 | 0.326 | 60% | 0.116 | R |
| EC | 1.20 | 0.75-1.91 | 0.444 | 79% | 0.030 | R |
| CC | 1.13 | 0.70-1.80 | 0.623 | 84% | 0.000 | R |
| NPC | **2.03** | **1.57-2.63** | **0.135** | 0% | 0.746 | F |
| BT | 1.25 | 0.45-3.48 | 0.674 | 91% | 0.001 | R |
| GC | 1.09 | 0.70-1.70 | 0.700 | 80% | 0.025 | F |
| **BB** vs. **AB+AA** | **1.17** | **1.04-1.31** | **0.009** | 43% | 0.010 | F |
| HWE | **1.18** | **1.05-1.32** | **0.005** | 41% | 0.014 | F |
| Asian | **1.17** | **1.06-1.30** | **0.003** | 23% | 0.172 | F |
| Caucasian | 1.01 | 0.63-1.61 | 0.979 | 65% | 0.014 | R |
| HCC | **1.17** | **1.00-1.37** | **0.045** | 0% | 0.476 | F |
| CRC | 1.11 | 0.86-1.44 | 0.422 | 0% | 0.712 | F |
| BC | 0.65 | 0.18-2.42 | 0.524 | 73% | 0.055 | R |
| EC | 1.23 | 0.76-2.01 | 0.399 | 75% | 0.046 | R |
| CC | 1.14 | 0.82-1.59 | 0.428 | 25% | 0.263 | F |
| NPC | **1.66** | **1.23-2.25** | **0.001** | 0% | 0.320 | F |
| BT | 1.00 | 0.43-2.30 | 0.993 | 80% | 0.026 | R |
| GC | 0.97 | 0.80-1.18 | 0.780 | 0% | 0.568 | F |
